# The apache ii and serum sodium levels as predictors of mortality in the surgical critically ill patients

**DOI:** 10.1186/2197-425X-3-S1-A757

**Published:** 2015-10-01

**Authors:** A Basile-Filho, MG Menegueti, EA Nicolini, AF Lago, EZ Martinez, M Auxiliadora-Martins

**Affiliations:** Department of Surgery and Anatomy, Ribeirão Preto Medical School, University of São Paulo, Ribeirão Preto, Brazil; Department Social Medicine, Ribeirão Preto Medical School, University of São Paulo, Ribeirão Preto, Brazil

## Introduction

The body sodium imbalance (dysnatremias) may be associated with increased mortality of critically ill patients. Evidence suggests that changes in the serum sodium level on admission to the intensive care unit (ICU), may lead to a poor outcome.

## Objectives

The objective of this study was to evaluate the ability of APACHE II and serum sodium levels to predict mortality of surgical critically ill patients.

## Methods

One hundred and ninety-five surgical patients (62 % males and 38% females; mean age of 51.8 ± 17.3 years) admitted to the ICU in the post-operative phase were retrospectively studied. The patients were divided into survivors (n=152) and nonsurvivors (n=43). APACHE II, and serum sodium levels at admission, 48-h and discharge were recorded. The capability of each index (APACHE II, Sodium-admission, sodium 48-h and sodium-discharge) to predict mortality of surgical patients was analysed by receiver-operator characteristic curves (ROC). Odds ratios (OR) and 95% confidence interval (CI) for hyponatremia (Na< 136 mmol/L) and hypernatremia (Na>144 mmol/L) were calculated.

## Results

**Figure 1:**
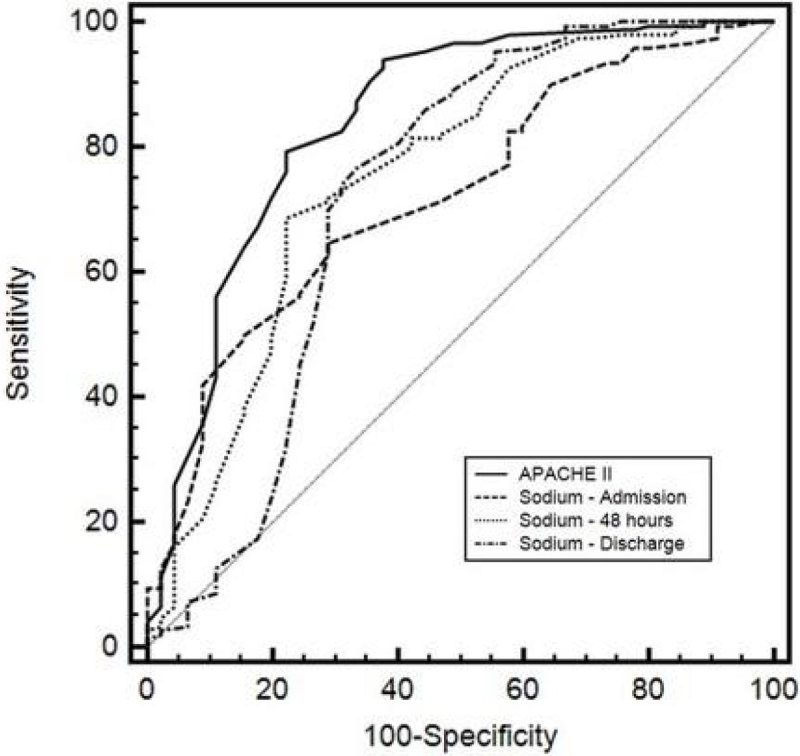
Figure 1

Comparison of data between survivors and nonsurvivors is summarized in Table 1. The mean APACHE II was 16.3 ± 8.3 (13.6 ± 6.1 for survivors and 25.5 ± 8.5 nonsurvivors). The area under the ROC curve for APACHE II was 0.841 (0.782-0.889) and 0.721 (0.653-0.783), 0.754 (0.687-0.812), 0.720 (0.651-0.782) for serum sodium level at admission, 48-h and discharge, respectively. OR for hyponatremia and hypernatremia were 5.33 (1.77-16.08) and 0.25 (0.1-0.65). The comparisons of ROC curves for these indexes are depicted in figure 1.

**Table 1:** Table 1

	Patients n = 195	Survivors n = 152	Nonsurvivors n = 43
Sex (M/F)	121/74	95/57	26/17
Age (years)	51.8 ± 17.1	51.5 ± 17.3	52.9 ± 16.8
APACHE II score	16.3 ± 8.3	13.6 ± 6.1	25.5 ± 8.5
Death risk (%)	25.3 ± 24.1	18 ± 17.3	50.6 ± 27.7
Mechanical ventilation (days)	2.6 ± 4.8	1.6 ± 3.6	6.13 ± 6.0
ICU length of stay (days)	4.3 ± 5.6	3.24 ± 3.8	8.2 ± 8.7
Hospital length of stay (days)	24 ± 21	23 ± 22	24 ± 20
Overall mortality (%)	22

## Conclusions

Even though APACHE II was the most effective index to predict mortality in the surgical critically ill patients, the serum sodium levels on admission may also be used as predictor of outcome.
